# Flowering Synchronization Using Artificial Light Control for Crossbreeding Hemp (*Cannabis sativa* L.) with Varied Flowering Times

**DOI:** 10.3390/plants14040594

**Published:** 2025-02-15

**Authors:** Gergő Somody, Zoltán Molnár

**Affiliations:** Albert Kázmér Faculty, Széchenyi István University, 9200 Mosonmagyaróvár, Hungary; molnar.zoltan@ga.sze.hu

**Keywords:** hemp breeding, speed breeding, hemp crossbreeding

## Abstract

Hemp (*Cannabis sativa* L.), one of the earliest domesticated crops, has diverse applications in textiles, construction, nutrition, and medicine. Breeding advancements, including speed breeding, accelerate genetic improvements in crops by optimizing environmental conditions for reduced generation times. This study employed greenhouse and field experiments to develop a proprietary yellow-stemmed hemp germplasm with a unique stem trait. Initial crossbreeding between the late Eletta Campana (medium green stems) and the early Chamaeleon (yellow stems) demonstrated the recessive monogenic inheritance of the yellow-stem trait and fast and safe stabilization even in the case of parent varieties with different flowering times. Controlled flowering in the case of photoperiod-sensitive genotypes, manual pollination, and successive backcrossing stabilized the yellow-stem trait over six cycles, with 100% trait consistency achieved by the fifth cycle within just 12 months in total. Open-field trials validated greenhouse results, showing strong correlations between visual stem color assessments and visible atmospherically resistant index (VARI) obtained through remote sensing imagery. Cannabinoid analyses indicated significant reductions in tetrahydrocannabinol (THC) content while maintaining optimal cannabidiol (CBD) levels. Accumulated growing degree days (GDDs) optimized flowering and maturity, ensuring consistency in phenological traits. This research highlights the utility of speed breeding and chemical analysis to accelerate trait stabilization and improve industrial hemp’s agronomic potential for fiber and CBD production while adhering to regulatory THC limits.

## 1. Introduction

Hemp (*Cannabis sativa* L.), one of the earliest domesticated agricultural crops, has been widely utilized for recreational, medicinal, and industrial purposes [1]. Its fibers serve as raw materials for the textile and construction industries, its seeds yield oil rich in highly unsaturated fatty acids, and cannabinoids extracted from its inflorescences fulfill both recreational and medicinal needs [2,3,4].

In the context of a growing global population and increasing challenges to crop productivity, speed breeding, or rapid generation cycling, has become highly relevant for advancing crop genetics. Originating from carbon arc lamp experiments over 150 years ago, speed breeding has evolved into a modern greenhouse technique employing LED technology and darkening systems to accelerate breeding cycles. This method complements approaches like single plant selection and single seed descent and shows great potential when combined with gene editing, genotyping, and genomic selection [5,6,7,8,9].

‘Speed breeding’ optimizes environmental conditions to shorten generation times in crops such as wheat, canola, and chickpea. Long-day plants, which require more than 16 h of light to flower quickly, benefit from this technique, while short-day plants like soybean need more complex protocols. Hemp, also a short-day plant, requires extended periods of daily darkness and only 11–15 h of light. This photoperiod dependency extends its generation time in the field, limiting breeding progress to just one generation per year [10,11,12].

*Cannabis sativa*, alongside *Humulus lupulus*, was among the first plants identified as having flowering regulated by photoperiod [13]. Considerable variation exists in flowering times among *C. sativa* cultivars, including day-neutral or ‘autoflowering’ varieties that are photoperiod-insensitive and capable of flowering under continuous light. These autoflowering cultivars, which mature more quickly and are generally shorter, enable cultivation in higher latitudes and controlled environments under long-day conditions [14].

The natural crossing of species [15] and varieties with differing flowering times has been extensively studied [16,17]. For example, research on the yellow-stemmed hemp phenotype traces its origins to Hoffmann’s 1946 discovery of the “Hellstengeligen” trait, which resulted from crossing Italian and Finnish landrace varieties [18]. This trait has become a focal point in hemp breeding programs, leading to the development and registration of several yellow-stemmed cultivars such as ‘Carmaleonte’, ‘Ivory’, ‘Kompolti Sárgaszárú’, ‘Fibror 79’, ‘Markant’, and ‘Chamaeleon’.

The primary goal of our research was to develop two low-THC, yellow-stemmed fiber hemp varieties with different maturity times and final plant heights. We aimed to achieve early-maturing, low-growing, yellow-stemmed plant material through targeted crossbreeding with the early-maturing, domestically adapted Balaton variety. Although this could have been accomplished purely under open-field conditions, it would have required significantly more time [19]. Yellow-stemmed hemp varieties are distinctly different from drug-type hemp, and we intentionally integrated this trait to help overcome legal challenges associated with hemp cultivation. At the same time, we remained committed to preserving the beneficial characteristics of the original parent varieties to create a yellow-stemmed variety similar to the famous Italian Eletta Campana, with a shorter generation time in order to seed production, which should also be feasible at our latitude. Furthermore, our goal was to develop an early-maturing variety with high seed yield potential that is easy to harvest. This variety also features a yellow stem while retaining the favorable traits of the parent variety, Balaton.

This study provides a detailed account of the breeding process initiated by crossing two varieties with markedly different maturity times and growth habits. The process incorporates the recessive yellow-stem trait, which can be easily evaluated in large populations. The study aims to assess the feasibility of accelerated breeding for this purpose and its potential role in conventional variety development based on open-field selection and progeny evaluation. The approach aligns with low-energy sustainability requirements while striving to adapt to the conditions of a rapidly changing cultivation environment.

Studies suggest that yellow-stemmed hemp offers significant advantages over green-stemmed varieties. These benefits include a higher proportion of long fibers relative to total fiber content, as well as finer bast fibers in processed bundles, making them highly desirable for industrial applications [20,21,22,23].

Moreover, previous studies [24,25,26] have aimed to explore the relationship between the qualitative and quantitative properties of medicinal and industrial hemp, both in completely artificial cultivation systems and under open-field conditions, promoting the broader applicability of marker-assisted and precision breeding for this plant species.

At the same time, the role of accelerated breeding in conventional breeding and variety development processes should not be underestimated. The previously published protocol [27] demonstrated the production of viable seeds within an extremely short timeframe. However, it yielded very few seeds, which may suffice for scientific investigations and molecular genetic research but does not provide a basis for potential variety development or subsequent open-field selection work. Furthermore, the operation of cultivation systems entirely reliant on artificial lighting is highly energy-intensive and requires a high level of technical sophistication. That said, the results are highly reproducible, and the system is easily standardizable.

Therefore, we aimed to create a model environment that maximally utilizes sunlight, even during periods unsuitable for hemp cultivation in our climate, while maintaining low energy consumption and relatively low infrastructure requirements. At the same time, our goal in this system was also to produce sufficient quantities of seeds suitable for progeny evaluation and open-field selection.

## 2. Results

### 2.1. Greenhouse Experiments

In 2021, a hemp breeding program was initiated with the goal of developing proprietary germplasm characterized by distinct morphological traits and population uniformity. The selection process prioritized high fiber yield and low tetrahydrocannabinol (THC) content, as expected from dioecious germplasm. A secondary selection aimed to maximize cannabidiol (CBD) content.

To achieve these goals, registered varieties were selected based on prior literature and cultivated in a greenhouse environment. Preliminary test crossings and progeny evaluations narrowed the focus to two varieties: the Italian Eletta Campana [28] from Canapuglia and the Dutch yellow-stemmed Chamaeleon from Wageningen University. Crossbreeding these varieties presented challenges due to their significantly different maturity times; Chamaeleon is an early-maturing variety, flowering in approximately 70 days, while Eletta Campana is late-maturing, flowering in 130 days.

A novel accelerated breeding method was developed to address this discrepancy and enable detailed morphological characterization, high seed yield, and identification of ideal individuals. A speed breeding protocol was introduced with shorter generation times [27], although our project started before the announcement of that protocol. Crossbreeding can be achieved with such a short generation time, but the essential distinguishing features are very different from field observations.

Spontaneous open-field crossing was impractical due to differences in anthesis timing. However, controlled flowering in a greenhouse enabled simultaneous crossing, as both varieties were photoperiod-sensitive.

Seeds were germinated in trays in a seedling room. Healthy seedlings suitable for transplanting were placed in the greenhouse.

Based on calculated growing degree days (GDDs), the plants that exhibited varietal characteristics and sufficient vegetative mass were exposed to short-day conditions using a blackout system that provided 12 h of continuous darkness. The sex of hemp individuals became clear within a week, as the initial distinguishing characteristics of the male and female individuals became visible, and flowering began by the 10th day. By the 14th day, artificial pollination was performed to ensure an adequate seed set. For Chamaeleon, both male and female plants were retained, while only female Eletta Campana plants were kept. During the summer, to avoid external pollen contamination, manual pollination was delayed until the pistils had grown unfertilized for a week.

The 12-h day length was maintained throughout the generative phase. When seeds with the desired color and marbling appeared, water stress was applied to accelerate ripening. The plants were harvested manually, and after threshing, the cannabinoid content of the waste was analyzed. Seeds from plants with low THC and high CBD content were re-sown. This process was repeated ten times over two years.

In the first filial generation (F1, second cycle), progenies from Eletta Campana × Chamaeleon (EL×CH) and Chamaeleon × Chamaeleon (CH×CH) crosses were evaluated. Five mother plants were selected from each cross, and 100 seedlings were transplanted as described. Evaluations were conducted using the CPVO protocol for tests on distinctness, uniformity, and stability. The main stem color was categorized as yellow (1), medium green (2), dark green (3), or purple (4). While Eletta Campana had medium green stems, Chamaeleon exhibited yellow stems. In the EL×CH progeny, no yellow-stemmed plants were observed, whereas all CH×CH plants displayed yellow stems, confirming the trait’s recessive monogenic inheritance.

For faster progress, EL×CH progenies were backcrossed with the Chamaeleon father. The CH×CH progeny reaffirmed the absence of external pollen contamination. In the second generation (third cycle), 400 seedlings from four EL×CH backcrossed mother plants were transplanted. At flowering, 183 plants had yellow stems, and 186 had green stems, while 31 plants were excluded due to underdevelopment or failure to emerge. Pollination was performed with yellow-stemmed males, resulting in 90 yellow and 95 green female plants at harvest.

In the fourth cycle, two elite plants were selected, and 354 seedlings were transplanted. Following pollination with a yellow-stemmed male pollen mixture and subsequent removal of the males, 153 female plants were harvested, 19 of which exhibited green stems. By the fifth crop cycle, all 150 elite plants from five selected females displayed yellow stems.

Subsequent breeding cycles focused on stabilizing additional parameters, including balanced flowering times, leaf morphology, seed yield, and CBD content. The yellow stem trait was successfully incorporated into the germplasm, as documented in Table 1, which summarizes the cycle dates and stem color observations.

### 2.2. Open-Field Experiments

In order to evaluate families with a larger sample size and to increase the amount of seed, field trials were also carried out. The aim of the field trials was to obtain a complete picture of the plant material, including its flowering time, spontaneous pollination and seed set capacity, real plant height, and adaptability to environmental conditions.

Seeds from 30 mother plants were sown from the propagated plant material. Ten individuals came from the first crossing (F1, cycle-1), ten plots were created from backcross seeds (BC1, cycle-2), and ten from the harvest of the first family breeding (cycle-3). The experiment was set up on 12 April 2022, in Hédervár, North-West Hungary, using a Wintersteiger plot seeder for sowing. The plots were 12 m^2^ in size, and seeds were sown at a depth of 3 cm, with a row spacing of 50 cm and a seed quantity of 45 plants/m^2^. In the case of five plots, the plant number was found to be low (<30/m^2^), so they were excluded from further investigation.

In order to further utilize the breeding material, the plots sown from the first cycle seeds were completely emasculated, while in the plots established as a result of the second and third cycles, only the yellow-stemmed male plants were left. Only the seeds of the yellow-stemmed female plants were harvested.

One hundred randomly chosen plants were assessed in each plot and the field observations showed a close correlation with the data from the previous year’s greenhouse experiments. A total of 94.57% of plants from the first family selection (harvested cycle-3 seeds) had yellow stems. In the case of the offspring from cycle-2, 50% were yellow-stemmed. No yellow-stemmed plants were observed on the plots of cycle-1 progenies.

In 2023, the stabilized families were sown in a small-plot experiment similar to the previous year in order to increase the seed quantity. The sowing conditions of the experiment were the same as in the previous field trial. A total of 45 plots were established from the different half-siblings of cycle-6, cycle-7, and the open-field trial. In this year, no green-stemmed individuals were found, so emasculation was performed based on other breeding parameters.

**Figure 1:**
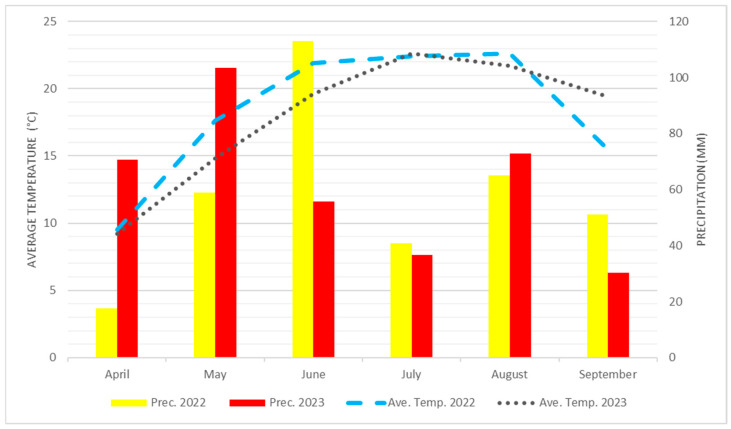
The precipitation and average temperature of 2022 and 2023.

The main meteorological parameters are illustrated in Figure 1.

In 2023, seeds from all cycles were sown in a small-plot trial to evaluate the morphological differences and homogeneity. The percent of yellow-stemmed plants was assessed at seed formation by visual observation of 100 randomly chosen plants per plot. In addition, we tried a preliminary UAV method, so aerial photos were taken at the seed formation of the Chamaeleon variety.

**Figure 2:**
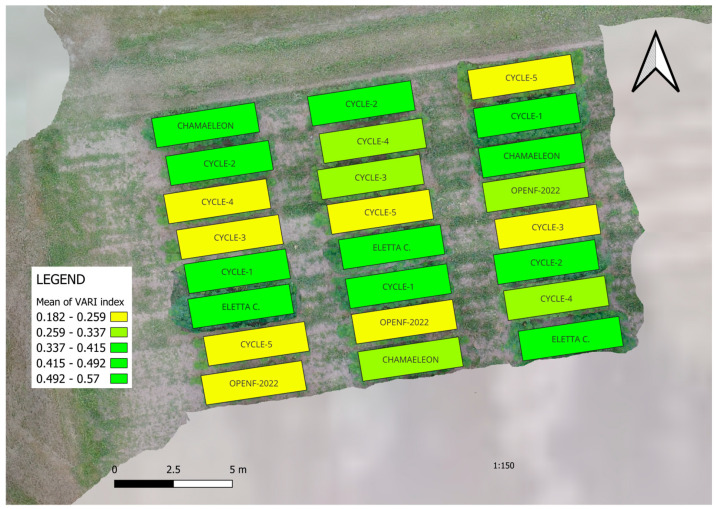
The calculated and visualized VARI indexes (Visible Atmospherically Resistant Index). Medium late ‘E’ selection from different speed breeding cycles (CYCLE-1: backcross with Chamaeleon males; CYCLE-2: 1st family selection; CYCLE-3: 2nd family selection; CYCLE-4: 3rd family selection; CYCLE-5: 4th family selection) were compared to the plots originated from the 2022 open-field trial and registered varieties Chamaeleon and Eletta campana.

In Figure 2, the calculated VARI indexes [29] from the aerial photos are observable after flowering. The Visible Atmospherically Resistant Index (VARI) is designed to emphasize vegetation in the visible portion of the spectrum while mitigating illumination differences and atmospheric effects. It is ideal for RGB or color images and utilizes all three color bands.

$$VARI=\frac{Green-Red}{Green+Red-Blue}$$

*Green* = pixel values from the green band;

*Red* = pixel values from the red band;

*Blue* = pixel values from the blue band.

The limitations of the VARI index and vegetation indices are well known [30]; in the present study, they serve a representational purpose to quantify visually distinguishable differences. There are a number of studies in the literature that utilize vegetation indices for leaf color evaluation [31,32,33].

Based on the data analysis results in Table 2, the stem color groups from VARI indexes are similar to the manual observations. However, the number of plants differed between plots, which affected the VARI index. It should also be noted that the yellow-stemmed plants have less intensive green leaf color, and they become yellower throughout the vegetation period. This trait may have been greatly influenced by our selection process. To avoid unwanted pollination, the more intensely green plants were removed before the change in stem color became clearly visible at the beginning of flowering.

There is a strong correlation (r = 0.8) between the two methods.

**Figure 3:**
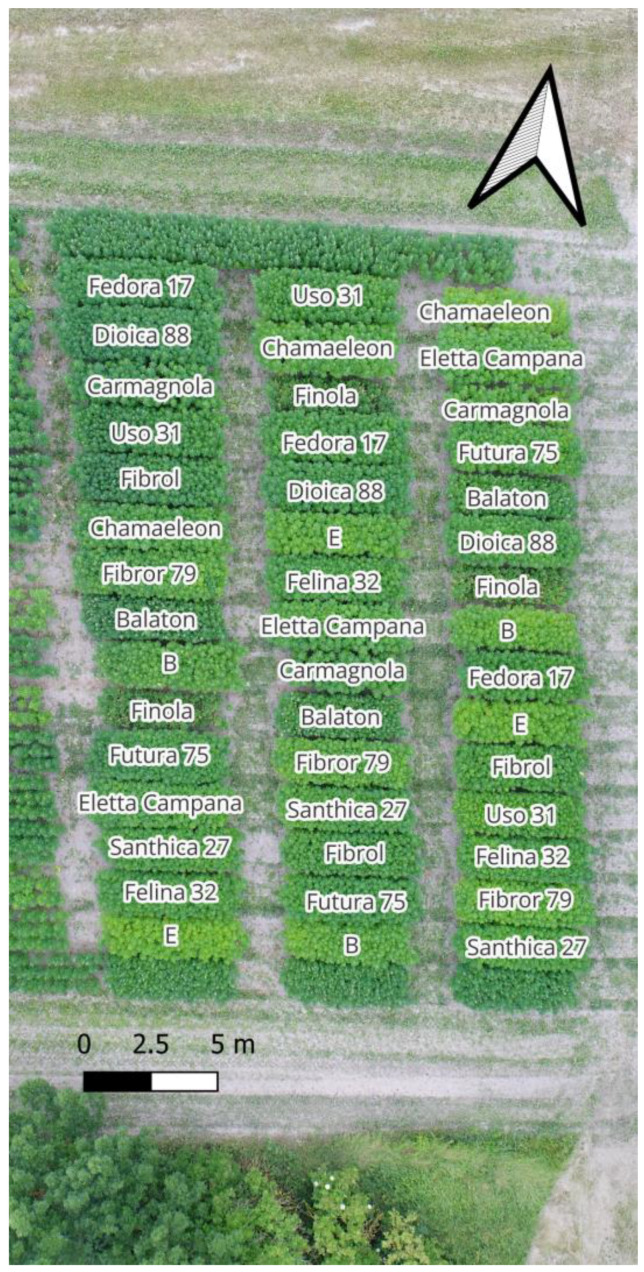
Aerial photo of the variety trial site in 2023. Medium late ‘E’ selection from CYCLE-5: 4th family selection and Early ‘B’ selection from CYCLE-5 were compared to registered varieties Balaton, Chamaeleon, Eletta Campana, Carmagnola, Dioica 88, Fedora 17, Felina 32, Fibrol, Fibror 79, Finola, Futura 75, Santhica 27, and USO 31.

In 2023, a variety comparison experiment was established to evaluate the traits of our newly developed varieties that need to be assessed in densely planted rows. The intensity of the green color of the leaves is a varietal characteristic. It must be examined during the evaluation of the variety. The leaves of yellow-stemmed varieties are less intensely green, regardless of the phenological stage. However, it is advisable to conduct the comparison before flowering. Figure 3 shows an aerial photograph of the variety trial.

The intensity of the green color in the leaves can be subjectively assessed at the plot level through visual evaluation; however, we compared the data derived from the VARI index with quantifiable data obtained from a SPAD (Leaf Soil-Plant Analysis Development) chlorophyll meter, as shown in Table 3.

**Figure 4:**
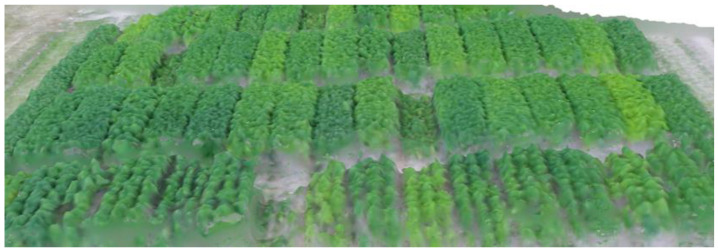
3D model of the variety trial constructed from aerial photos.

A digital surface model (DSM), a digital terrain model (DTM), and a 3D model (Figure 4) were also created from the aerial photographs. In the experiment sown with a high plant density, a strong correlation (r = 0.93) was observed between the estimated and measured plant height values.

### 2.3. Cannabinoid Content

During the whole breeding period, the cannabinoid content of the plants was evaluated. The growability of hemp is largely determined by its THC content, which in Hungary, is set at a maximum value of 0.2%. We used a cheap and fast TLC method to screen a large number of mother plants, and we carried forward the seeds of plants with the lowest possible THC and the highest possible CBD content. We used a ready-to-use test kit (Alpha-Cat TLC kit from Canebe SRO).

In 2023, the cannabinoid content was again measured.

Table 4 shows that the selection of the initial crossbreeding material is very important. Individual selection based on chemical parameters was only introduced from the second cycle onward.

### 2.4. Natural and Artificial Flowering and Maturity

Our primary goal was the development of open-field varieties; therefore, we aimed to compare our greenhouse observations in some form with the characteristics observed under open-field conditions, hoping that this would facilitate the selection process.

As Table 5 shows, 78–93 days passed between seeding and harvesting in the case of the Eletta Campana × Chamaeleon (E) Cycle-4, -5, and -6 greenhouse vegetation cycles. The accumulated GDDs varied between 2013 and 2397 GDDs calculated from the average temperature records without modification. The lengths of the cycles were 83–91 days in the case of the Balaton × Chamaeleon (B) selection.

There is a variation between the phenological stages of individual plants mainly because there is a large difference in morphology and ripening time between the parent varieties.

The early or autoflowering and too-late plants were removed, because our main goal was stabilization and open-field multiplication.

The optimal time for switching to short-day conditions was the date when around 1000 GDDs had been accumulated. In our system, the necessary vegetative mass and ideal final plant height were observable after this period.

Using the Mediterranean phenological prediction literature [34,35,36], the modified GDD values can also be calculated. ‘Tiborszállási’ is a Hungarian variety with similar morphological and growth characteristics, precisely because of this, the GDD value, or thermal sum (°C d) was calculated from the following equation:

$$Cd\;\left(GDD\right)=\sum_{i=1}^{n}\frac{\left({T_{min}}_{i}-T_{b}\right)+(T_{{\prime max}_{i}})}{2}$$

where *n* = number of days to the end of vegetation; $T_{{min}_{i}}$ = minimum temperature at day *i*; $T_{b}$ = base temperature (°C) under which the plant development stops, regarding Tiborszállási this value is found to be 1.9 °C. $T_{{\prime max}_{i}}$ = modified maximum temperature at day *i*, if $T_{max}\leq T_{opt}$ then $T_{{\prime max}_{i}}=T_{max}$, if $T_{max}>T_{opt}$ then $T_{{\prime max}_{i}}=T_{opt}-(T_{{\prime max}_{i}}-T_{opt})$ $T_{opt}$ is the optimum temperature for plant development, in the case of Tiborszállási it is found to be 25 °C.

The modified GDD values with base and optimum temperatures were added to the table; however, in our case, the calculation with the modified values would not have been beneficial; especially in early spring, values are overestimated compared to the real photosynthetic activity and visible green mass growth. However, in the case of July–August, when the irradiation experienced can be simulated with an artificial light source, the calculation with modified values may be more reliable.

In comparison with open-field data in 2022, the length of growing season was 140 days, in 2023. A total of 153 days passed from sowing to harvest. This represents a selection of a mid-late maturity group.

The earlier selection (B) was harvested 129 days after sowing in 2022 and 140 days after sowing in 2023. Table 6 summarizes the relevant open-field observations.

### 2.5. Yield Comparison

The quantity of harvestable seed yield, along with its usability and quality indicators related to variety characteristics, are important factors in evaluating the method. In greenhouse cultivation, the seed yield of mother plants selected based on morphological criteria that can also be compared to their open-field appearance must be sufficient to establish field plots and, due to genetic progress, potentially function as pollen donors across the entire breeding nursery. Additionally, in speed breeding, the male-flowering individuals from progeny originating from mother plants that exceeded threshold values (cannabinoid content, seed yield, plant height) and exhibited the desired traits (stem color, stem diameter, leaf morphology, estimated maturity time, thousand-seed weight, seed color, and marbling) were designated as pollen donors.

**Figure 5:**
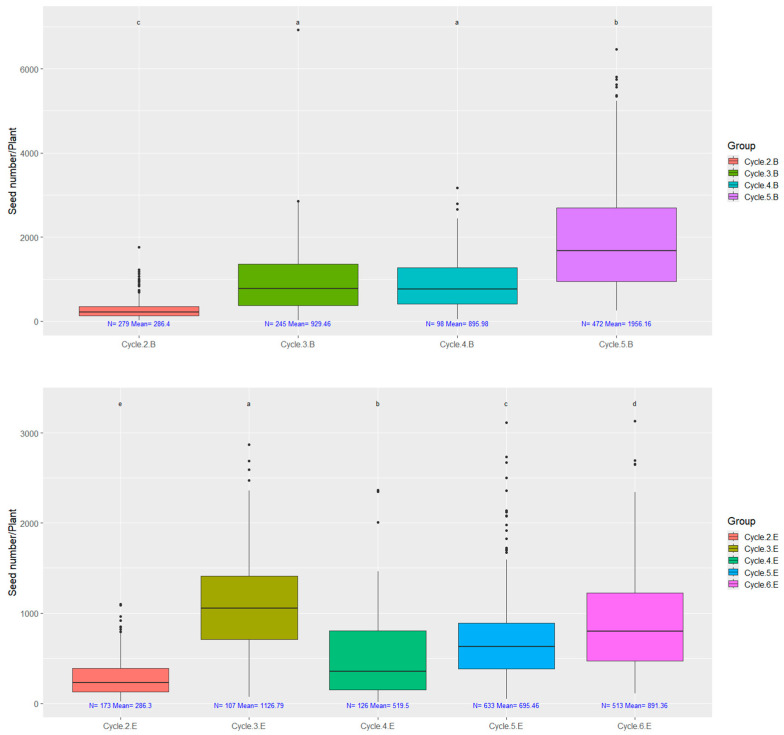
The number of seeds from individual plants in case of both selections. Data ranges counted from rapid generation cycling of early ‘B’ strain cycle-2; cycle-3; cycle-4; cycle-5 and medium-late ‘E’ cycle-2; cycle-3; cycle-4; cycle-5; cycle-6. On top of each boxplot, groups labeled with the same letter do not significantly differ, as determined by both ANOVA followed by Tukey’s HSD and Welch’s ANOVA followed by the Games–Howell post hoc test (*p* = 0.05).

The number of seeds harvested from individual plants is shown in Figure 5.

**Figure 6:**
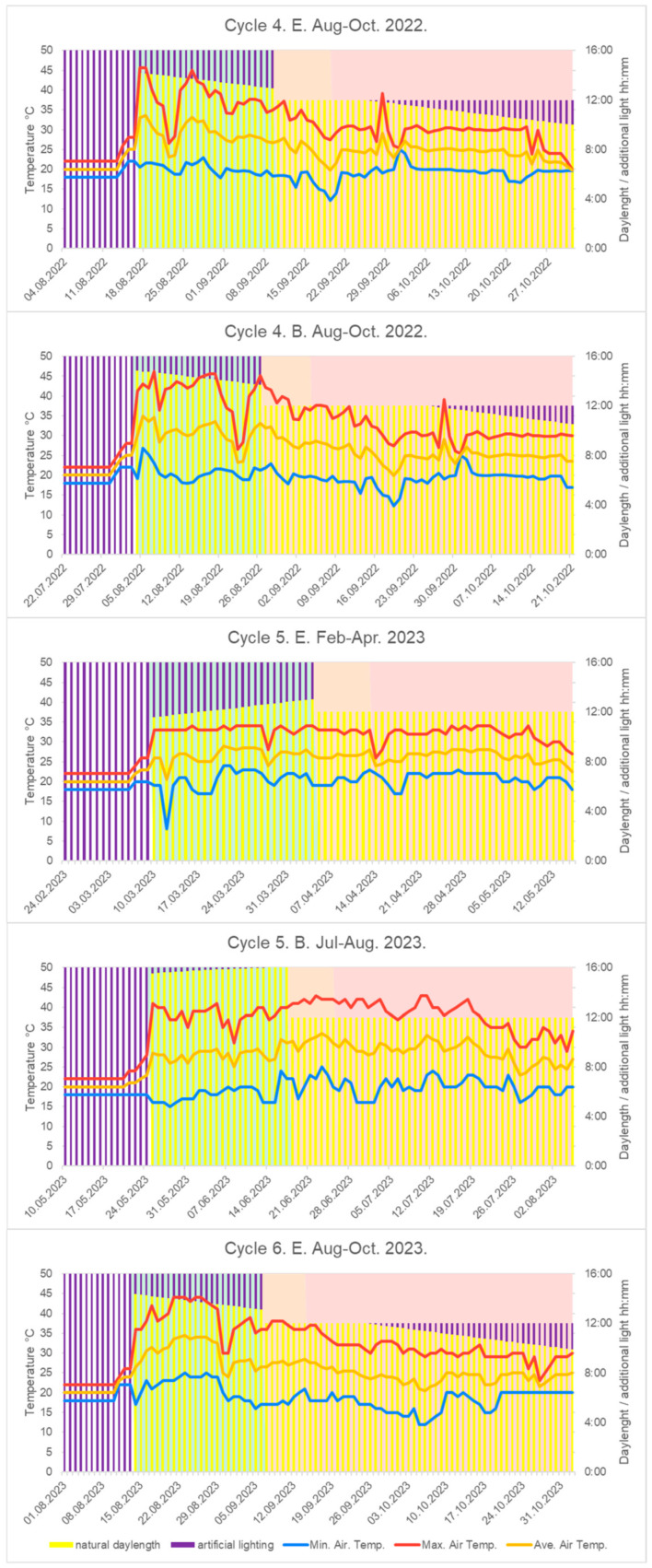
Natural daylength, additional artificial lightning, and air temperature records under rapid generation cycling (Cycle-4, -5, -6). ‘E’ represents the medium-late-maturing selected family, while ‘B’ refers to the early selection.

To evaluate seed yield, it is important to examine the climatic factors of greenhouse cultivation (Figure 6).

**Figure 7:**
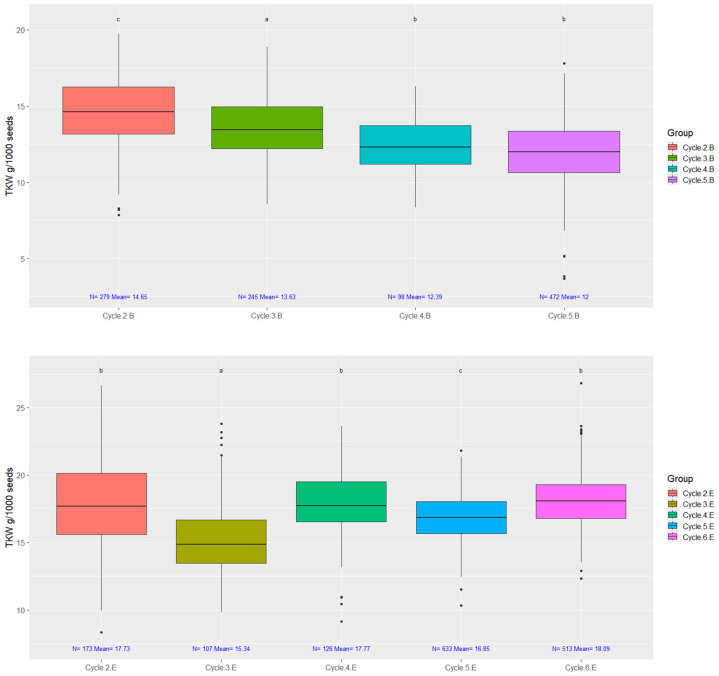
The thousand kernel weight (TKW) of seeds from individual plants in case of both selections. Data ranges counted from rapid generation cycling of early ‘B’ strain cycle-2; cycle-3; cycle-4; cycle-5 and medium–late ‘E’ cycle-2; cycle-3; cycle-4; cycle-5; cycle-6. On top of each boxplot, groups labeled with the same letter do not significantly differ, as determined by both ANOVA followed by Tukey’s HSD and Welch’s ANOVA followed by the Games–Howell post hoc test (*p* = 0.05).

Thousand kernel weight (TKW) is an important parameter and a varietal characteristic; therefore, it served as a selection criterion. Figure 7. shows the changes in TKW during the rapid generation cycling.

**Figure 8:**
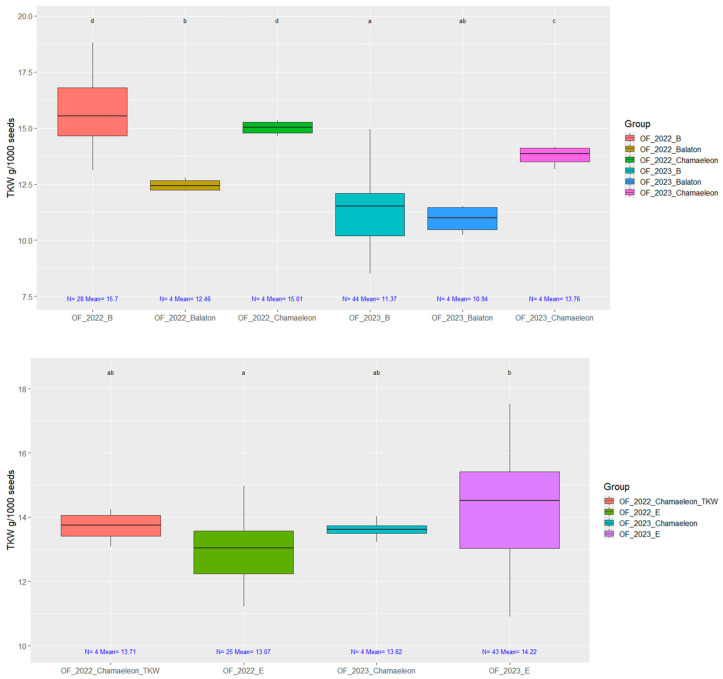
The thousand kernel weight (TKW) of seeds was measured from whole plots in open-field experiments. Data ranges were visualized from open-field trials conducted in 2022 and 2023. In these trials, the two seasons of early ‘B’ selection were compared to the registered varieties *Chamaeleon* and *Balaton*. Additionally, the medium-late ‘E’ selection from both years was compared only to *Chamaeleon* in a separate experiment. On top of each boxplot, groups labeled with the same letter do not significantly differ, Welch’s ANOVA followed by the Games–Howell post hoc test (*p* = 0.05).

We examined the seed yield and thousand kernel weight harvested from open-field plots in two completely different open-field growing seasons (Figure 8).

Regarding the initial material, the thousand kernel weight of the Balaton variety used for the first crossing was 14.2 g, while for Eletta Campana, TKW was 20.3 g. In both cases, the Chamaeleon variety was also used, with a thousand kernel weight of 16.2 g. Our main goal was to maintain the high TKW for the medium-late ‘E’ selection. In the case of the early ‘B’ selection, the seed yield was much more important, so the more compact mother-like (Balaton-type) individuals remained and propagated further.

**Figure 9:**
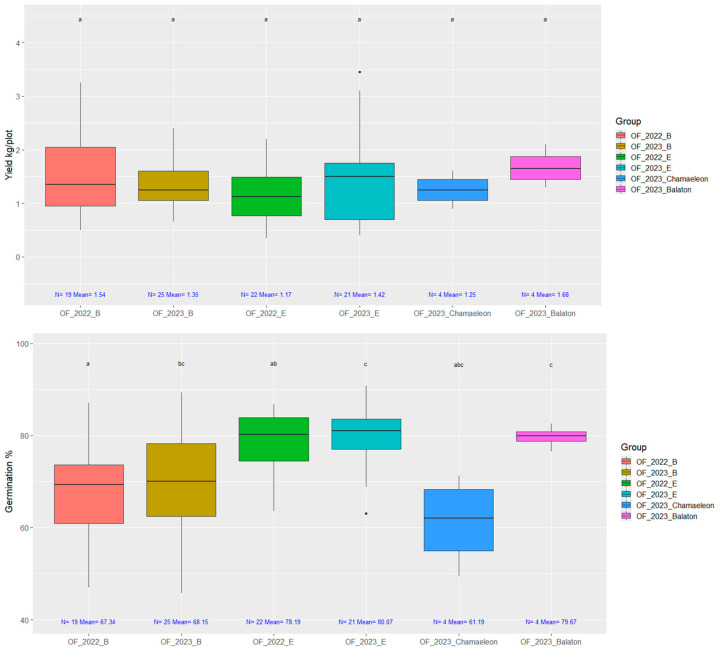
The yield and germination% of the 2023 open-field trial. OF_2022_B refers to the early ‘B’ plots that originated from the 2022 open-field trial. OF_2022_E refers to the medium-late ‘E’ that originated from the 2022 open-field trial. OF_2023_B and OF_2023_E plots were sown from the speed breeding material. OF_2023_Balaton and OF_2023_Chamaeleon are registered varieties. On top of each boxplot, groups labeled with the same letter do not significantly differ, as determined by Welch’s ANOVA followed by the Games–Howell post hoc test (*p* = 0.05).

Figure 9 presents the seed yield and germination capacity of the plots sown in the 2023 field experiment. For comparison, plots were also included with seeds harvested from the 2022 field trials (OF_2022_B and OF_2022_E). The OF_2023_E and OF_2023_B plots originate from the previous cycle of the accelerated breeding process.

## 3. Discussion

The research presented here underscores the advancements in hemp breeding through the integration of speed breeding techniques, genetic selection, and environmental control in order to transfer recessive traits between varieties with quite different flowering times. Key findings from this study highlight the effective stabilization of the yellow-stem trait, significant progress in breeding for low THC and high CBD content, and the potential for modernized phenotyping through UAV technology.

Additionally, a sufficient quantity of seeds can be produced from the mother plants for field-scale propagation. By maximizing the use of natural light, the system’s energy demand remains relatively low compared to fully artificial lighting systems. Furthermore, heating is provided by a biomass-fired boiler, allowing for the utilization of byproducts from seed production.

### 3.1. Integration of Speed Breeding in Hemp Cross-Pollination

Since hemp is naturally an obligate cross-pollinating plant, the use of male individuals in traditional breeding is challenging, often resulting in extremely slow genetic progress. Many important qualitative and quantitative traits cannot be evaluated in male plants. In our case, pollination was conducted using individuals that developed from seeds of well-defined maternal plants. In the following year’s field nurseries, elite plots derived from accelerated breeding ensured pollination.

A crucial requirement for this process is that thousands of seeds must be available from a single plant for trials and field plot establishment (Figure 5). This allows for up to four selection cycles within a single year, which are subsequently evaluated and propagated under field conditions. Our data indicate a noticeable increase in seed quantity adapted to this system. In our breeding process, there was no significant correlation between the seed quantity per plant and the thousand kernel weight (r < 0.2). (Supplementery Table S1) Therefore, this trait was evaluated as a variety-specific characteristic, independent of environmental factors, which was confirmed by the field assessment (Figure 6).

The key question was whether this negatively impacts field cultivability. As shown in Figure 9, this type of selection does not adversely affect yields or the germination capacity of the final strains.

No fertilizers, pesticides, or irrigation were used in the field trials. A critical breeding objective was to respond to the increasing occurrence of heat waves and extremely hot days [37], which are becoming more restrictive for crop production in Hungary. Figure 5 highlights that in most cultivation cycles, maximum temperatures exceeded 40 °C. This had a particularly strong selective pressure during seedling transplantation, influencing their regeneration capacity. The field validation of this induced selection process will require several more years.

The speed breeding method used to accelerate crop improvement proved instrumental in this research. By manipulating photoperiods and environmental conditions, the breeding cycles were condensed, enabling up to ten generations over two years. This method was particularly crucial for overcoming the photoperiod dependency of hemp, a short-day plant, which traditionally limits breeding to one generation annually. The results demonstrate how speed breeding can be applied not only to long-day crops but also to photoperiod-sensitive crops like hemp when combined with controlled environmental settings.

### 3.2. Stabilization of Yellow-Stem Trait

The stabilization of the yellow-stem trait, which was inherited recessively, was achieved over multiple breeding cycles. The findings align with previous literature suggesting that yellow-stemmed varieties offer industrial advantages due to finer bast fibers and higher long-fiber content. By the fifth cycle, the yellow-stem trait had reached complete fixation within the selected population, marking a significant milestone in the breeding program. Open-field trials validated greenhouse observations, showing a consistent 100% occurrence of the yellow-stem trait in later cycles.

### 3.3. Advances in Phenotyping Techniques

A novel aspect of this study was the use of UAV-based Visible Atmospherically Resistant Index (VARI) measurements to differentiate between yellow- and green-stemmed plants. UAV phenotyping provided a cost-effective and efficient alternative to manual assessments, especially in large-scale field trials. However, challenges such as plant density differences and environmental variability affecting the VARI index were noted. Future research could refine UAV methodologies for improved accuracy and scalability. Height prediction is a very useful tool to evaluate breeding material, however interpreting results at every phenological stage in hemp is challenging, as the natural height of male and female plants differs. As noted in [38], height estimation is significantly affected by improper plant density, which was also observed in our case.

### 3.4. Cannabinoid Profiling

The study highlights the importance of selecting low-THC genotypes to comply with legal regulations. Initial crosses revealed high variability in cannabinoid content, with subsequent cycles showing a steady reduction in THC and stabilization of CBD levels. The selection process benefitted from chemical screening methods like the cost-effective TLC and advanced verification through AOAC official methods, demonstrating a robust and cost-effective approach to chemical phenotyping.

### 3.5. Natural and Artificial Maturing

In a controlled environment, the flowering time is directed with photoperiod manipulation. This was necessary for managing the final plant height, which is a limiting factor. The LED lights were placed at a height of 4 m, so plants must remain below this level. In the case of longer vegetative phases, overgrowth and plant damage were frequent.

For a more relevant growth analysis, the system should model the natural solar radiation typical for July and August. Our LED light system was designed only for supplementary lighting purposes. So the photoperiod manipulation was more safely based on absolute temperature values, which correlated better with observed growth vigor and expected maximum plant height even if the temperature rose above the optimum.

However, our breeding material was transplanted in every cycle to the greenhouse, which affects the length of the required vegetative stage. The regeneration time is the shortest in early spring. During this period it is easy to achieve rapid acclimatization and due to the constantly increasing radiation, ripening is faster. It is more difficult to find an ideal planting time in August as heat stress is greater.

Considering our crosses, the male parent was the early Chamaeleon variety and the female parent was the late Eletta Campana variety.

Based on open-field observations, it was possible to select strains from the mid-late maturity group, which is still capable of carrying a trait, similar to the female parent variety. The crossbreeding offspring predominantly belonged to this group. Seed formation and ripening are safe even at our latitude (N 47.8°).

For the sake of comparability, we conducted growing degree day (GDD) calculations to estimate how well the field characteristics of plant material selected under controlled conditions—such as plant height and flowering time—can be predicted. In the case of the early ‘B’ selection, the fundamental parameters were comparable, and the expected flowering and maturation times aligned with predictions. However, in the case of the mid-late ‘E’ strain, we drastically shortened the vegetative phase. In this case, the final plant height and maturation time were difficult to estimate.

### 3.6. Challenges and Limitations

Crossbreeding varieties with differing flowering times required precise environmental controls, particularly for photoperiod-sensitive traits. Ensuring genetic purity in both greenhouse and field settings demanded manual interventions, including emasculation and controlled pollination. Open-field trials exposed plants to climatic fluctuations, potentially influencing phenotypic expression and yield.

In a greenhouse environment, the evaluation of some important traits (natural maximum plant height, stem diameter, type of stem cross-section, maturity group) is difficult.

A relatively narrow set of traits was examined with highly specific objectives, so the general feasibility of the method for addressing multiple complex traits cannot be determined. Field trials were conducted in two drastically different growing seasons, and multi-location field cultivation is still ongoing. The effectiveness of the selection and breeding methods can only be evaluated after several years. Moreover, while large-scale, long-term field-based mother plant selection remains essential for genetic progress and yield stability, speed breeding enables the targeted incorporation of a few well-defined traits.

### 3.7. Industrial and Agricultural Implications

The successful incorporation of desirable traits such as a yellow stem, balanced flowering times, and a controllable cannabinoid profile demonstrates the commercial potential of this breeding program. The yellow-stemmed hemp varieties can be clearly distinguished from drug-type hemp, and we aimed to incorporate this trait to also address the legal barriers that hinder hemp cultivation. At the same time, preserving the favorable traits of the initial parent varieties was also a determined goal.

These improvements are directly relevant to the textile and pharmaceutical industries, offering high-quality raw materials with tailored cannabinoid profiles. Moreover, the adaptation of hemp to northern latitudes and controlled environments enhances its viability as a crop in diverse agroecological settings.

### 3.8. Future Directions

Chemotype inheritance and genetic markers for CBD and THC production are well studied [39,40]. Future research should focus on employing genomic tools to further elucidate the inheritance of critical recessive traits such as a yellow stem and other key quality features such as seed marbling and seed color.

Accurate climatic models and GDD calculations are necessary for every maturity group with expanded measured data (humidity, solar radiation) in order to predict the field performance of plant material produced through accelerated breeding.

For indoor hemp cultivation, it is essential to determine the energy requirements for lighting, heating, and cooling to meet the optimal quantitative and qualitative criteria for sustainability and production efficiency. Lighting is a well-researched topic with significant energy demands. Numerous studies have been published in this field [41,42], highlighting the importance of comprehensive growth analysis. Such research is crucial for assessing the industry’s carbon footprint and long-term sustainability.

For photoperiod-sensitive varieties, maintaining the vegetative stage does not require 16 h of continuous lighting. It is sufficient to limit the uninterrupted dark period to a maximum of eight hours by introducing short light interruptions, such as one-hour illumination periods. Even saving just one hour of lighting time can lead to a significant reduction in energy consumption. However, a comprehensive evaluation of its impact on vegetative biomass production is necessary.

Shading systems have relatively low energy demands in existing setups, making their use a relevant research topic for reducing cooling and heating energy consumption. Creating an optimally controlled cultivation environment that benefits both plant growth and sustainability is a crucial challenge of our time.

In a controlled environment, there is great potential for automation. Leveraging AI and advanced imaging techniques to refine phenotyping accuracy, handle very large plant breeding material, and replace subjective evaluation methods.

In the case of UAV-based screening methods, the application of AI offers significant advantages. As noted in a previous study [43], deep learning can help correct errors by providing a fast and efficient means of identifying individual plants, estimating plant height, assessing green color intensity, and detecting male-flowering individuals.

Testing stabilized varieties under diverse field and climatic conditions to assess adaptability and resilience to environmental stressors is very important in order to create conventional agricultural plant varieties.

This study represents a significant step forward in modern hemp breeding, demonstrating the potential of combining traditional methods with cutting-edge technology to meet the demands of industrial applications and regulatory frameworks.

## 4. Materials and Methods

### 4.1. Greenhouse Experiments

The trials in controlled environmental conditions took place in a 200 m^2^ double-layered, heated, ventilated polytunnel in Hédervár, Hungary. The automatic ventilation system is temperature-controlled. This was supplemented with 2 greenhouse ventilators to ensure uniform air temperature.

The artificial light control system contained a blackout system with an automatic time switch and 30 LED, three-channel plant growing lights (Parfactworks ZE250; PAR_max_ 1105 µmol/m^2^/s from 30 cm).

Each seedling was grown in a seedling nursery room in 170 cm^3^ of Pindstrup Plus Blue substrate (pH 6.0, 0–10 mm) without added fertilizer at a constant temperature of 18–22 °C under artificial LED lighting with 16 h photoperiods. Bottom irrigation was used to promote root development, maintaining a moist substrate surface.

When the seedlings reached 13 cm in height, they were transferred to the greenhouse for three days before being transplanted into 20 L containers filled with Pindstrup Mix + Clay substrate (pH 6.0, 10–30 mm). Two plants were placed in each container to ensure sufficient numbers after removing male plants.

A drip irrigation system was installed. After acclimatization, the watering schedule was supplemented with soluble fertilizers from a 1 m^3^ tank. Fertilizer recipes are given in Table 7. Watering varied depending on the rate of evaporation. Acidity was adjusted to pH 5.5.

Thermometers were placed in 6 different locations at a height of 1.2 m. The data reported are the averages of the 6 measurements.

Harvest was performed by hand. Seeds were threshed with a Wintersteiger LD350 laboratory thresher.

### 4.2. Open-Field Trials

Open-field trials in 2022 and 2023 were performed near Hédervár, Hungary using a Wintersteiger plot seeder for sowing. The plots were 12 m^2^ in size, and seeds were sown at a depth of 3 cm, with a row spacing of 50 cm and a seed quantity of 45 plants/m^2^. Trials were only rain-fed. Harvests were performed by hand, the sheaves dried next to build-up stands near the field. Seeds were threshed with the laboratory thresher.

### 4.3. UAV Image Creation and Processing

Images were captured on the 21 June 2023 using a DJI Phantom 4. The UAV was equipped with a DJI FC6310 camera (flying height above ground: 24 m).

Raster analysis and vector zonal statistics were performed using QGIS 3.32.2-Lima.

DEM (digital elevation model) and 3D models were created using OpenDroneMap/WebODM. (https://github.com/OpenDroneMap/WebODM) (accessed on 12 February 2025).

Statistical analysis (ANOVA, correlation) was performed using ARM Trial Management Software 9.1.0 (Gylling Data Management).

SPAD measurement was carried out using a Konica Minolta SPAD-502 Plus.

### 4.4. Comparison Graphs and Statistics

Anova, post hoc tests, and bar plots were created with R-4.4.2 for Windows with ‘multcompView’, ‘ggplot2’, ‘car’, ‘tidyr’, and ‘dplyr’ packages. In cases where the assumption of homogeneity of variances was violated, Welch’s ANOVA was used along with the Games–Howell post hoc test from the ‘rstatix’ package. For correlation analysis and visualization, the ‘Hmisc’ and ‘corrplot’ libraries were used.

### 4.5. Cannabinoid Analysis

In the case of the greenhouse breeding material, ready-to-use TLC kits were used (Alpha-Cat TLC kit from Canebe SRO).

The official, final evaluation of cannabinoid content in the open-field trials conducted in 2023 was performed by the Hungarian National Food Chain Safety Office (AOAC Official Method of Analysis 2018.11). Samples were collected according to the European Union’s recommended sampling procedure [44].

## Figures and Tables

**Table 1:** Stem color observations during the speed breeding process.

No. of Cycle	Period	Origin of Plants	Breeding Method	No. of Harvested Females	Yellow Stemmed %
1.	May–Aug. 2021.	Eletta Campana females—commercial seed	Cross with Chamaeleon males	300	Eletta Campana: 0% Chamaeleon: 100%
2.	Aug.–Nov. 2021	5 half-siblings of EL×CH Cycle 1	Backcross with Chamaeleon males	100	EL×CH: 0% Chamaeleon: 100%
3.	Jan.–Mar. 2022.	4 half-siblings of (EL×CH)×CH Cycle 2	Family selection	185	48.6%
4.	Apr.–May. 2022.	2 half-siblings Cycle 3	Family selection	153	87.6%
5.	Aug.–Oct. 2022.	5 half-siblings Cycle 4	Family selection	150	100%
6.	Feb.–May. 2023.	5 half-siblings Cycle 5	Family selection	649	100%
7.	Aug.–Nov. 2023.	2 half-siblings Cycle 6	Family selection	532	100%

**Table 2:** Statistical differences were analyzed between VARI (Visible Atmospherically Resistant Index) values derived from aerial images and the manually counted yellow-stemmed plants in the 2023 trial. The plots were established from seeds originating from different speed breeding cycles (CYCLE-1 to CYCLE-5) and the previous year’s open-field growing season (OPENFIELD-2022). Additionally, Eletta Campana and Chamaeleon were the registered varieties.

	Origin of Seeds	VARI Index	Sig. Group *	% of Green-Stemmed Plants	Sig. Group *
1	CYCLE-1	0.42	ab	100.0	a
2	CYCLE-2	0.36	abc	52.0	b
3	CYCLE-3	0.25	c	0	c
4	CYCLE-4	0.28	bc	0	c
5	CYCLE-5	0.24	c	0	c
6	OPENFIELD-2022	0.25	c	0	c
7	ELETTA CAMPANA	0.46	a	100.0	a
8	CHAMAELEON	0.34	abc	8.3	c
Tukey’s HSD (*p* = 0.05)		0.166		9.44	
Standard Deviation		0.058		3.28	
CV		17.78		10.07	

* Means followed by the same letter do not significantly differ (*p* = 0.05, Tukey’s HSD).

**Table 3:** Statistical differences in VARI indexes from the aerial images and the measured SPAD values.

		SPAD Value		VARI Index	
No.	Variety	Mean	Sig. Group *	Mean	Sig. Group *
1	B	31.63	e	0.37	de
2	Balaton	40.53	a	0.50	a
3	Carmagnola	37.40	abc	0.47	a–d
4	Chamaeleon	32.70	de	0.38	cde
5	Dioica 88	39.77	ab	0.52	a
6	E	31.73	e	0.35	e
7	Eletta Campana	35.80	cd	0.43	a–e
8	Fedora 17	37.70	abc	0.52	a
9	Felina 32	35.60	cd	0.47	a–d
10	Fibrol	37.23	abc	0.52	a
11	Fibror 79	31.43	e	0.39	b–e
12	Finola	38.70	abc	0.38	b–e
13	Futura 75	36.63	bc	0.49	ab
14	Santhica 27	35.17	cde	0.45	a–e
15	Uso 31	36.97	abc	0.48	abc
Tukey’s HSD (*p* = 0.05)		3.765		0.106	
Standard Deviation		1.243		0.035	
CV		3.46		7.79	

* Means followed by the same letter do not significantly differ (*p* = 0.05, Tukey’s HSD).

**Table 4:** Analysis results of the main cannabinoids.

Origin of Seeds for Small Plot Trial	CBD mg/kg N = 3		CBDA mg/kg N = 3		Δ^9^-THC mg/kg N = 3		THCA mg/kg N = 3	
Cycle-1	15,486	±3097	13,956	±2791	2241	±448	1414	±283
Cycle-2	16,612	±3322	7832	±1566	577	±115	193	±38.6
Cycle-3	14,156	±2831	13,870	±2774	369	±73.8	159	±31.8
Cycle-4	13,059	±2612	8882	±1776	329	±65.7	153	±30.5
Cycle-5	13,734	±2747	10,800	±2160	374	±74.9	122	±24.4
Cycle-6	14,224	±2845	12,200	±2440	293	±58.6	99.8	±20
Openfield–2022	19,295	±3859	13,366	±2673	690	±138	170	±34.1
Eletta Campana	46,856	±9371	26,455	±5291	532	±106	54.7	±10.9
Chamaeleon	16,191	±3238	13,289	±2658	391	±78.3	76.3	±15.3

**Table 5:** Length of the main phases and accumulated GDD (Growing Degree Day) values of Cycle-4, -5, and -6 of the speed breeding process. ‘E’ represents the medium-late selection; ‘B’ refers to the early-maturing breeding material.

Date	Event	Days from Sowing		Accumulated Growing Degree Days		Modified Growing Degree Days	
	Cycle-4–E (N = 126)						
17 August 2022	transplanting	13	±0	306	±0	199	±0
10 September 2022	change photoperiod to 12/12	37	±0	997	±0	556	±0
20–25 September 2022	full male flowering	49	±2	1298	±62	764.5	±45.5
28–31 October 2022	harvest	87	±2	2200.5	±31.5	1491.5	±9.5
	Cycle-5–E (N = 633)						
10 March 2023	transplanting	14	±0	314	±0	214	±0
5 April 2023	change photoperiod to 12/12	40	±0	1007	±0	670	±0
13–17 April 2023	full male flowering	50	±2	1270.5	±49.5	852	±38
13–15 May 2023	harvest	79	±1	2036.5	±23.5	1389.5	±19.5
	Cycle-6–E (N = 513)						
14 August 2023	transplanting	13	±0	298	±0	195	±0
7 September 2023	change photoperiod to 12/12	37	±0	1023	±0	518	±0
17–20 September 2023	full male flowering	48	±2	1337	±39	731.5	±25.5
1–2 November 2023	harvest	92	±1	2384.5	±12.5	1512.5	±9.5
	Cycle-4–B (N = 98)						
4 August 2022	transplanting	13	±0	306	±0	257	±0
27 August 2022	change photoperiod to 12/12	36	±0	1015	±0	591	±0
5–12 September 2022	full male flowering	49	±3	1369	±95	782	±54
17–21 October 2022	harvest	89	±2	2374.5	±48.5	1516.5	±36.5
	Cycle-5–B (N = 472)						
24 May 2023	transplanting	14	±0	307	±0	289	±0
18 June 2023	change photoperiod to 12/12	39	±0	1015	±0	639	±0
22–26 June 2023	full male flowering	45	±2	1203.5	±63.5	721	±28
1–5 August 2023	harvest	85	±2	2360	±51	1327.5	±35.5

**Table 6:** Key phenological observations and accumulated Growing Degree Day (GDD) values from open-field trials in 2022 and 2023. ‘E’ represents the medium-late-maturing selection, while ‘B’ refers to the early-maturing breeding material.

Event	Days from Sowing				Accumulated Growing Degree Days		Modified Growing Degree Days	
	**2022 (n = 250)**							
	**E.**		**B.**		**E.**	**B.**	**E.**	**B.**
emergence	5	±1	5	±1	82	82	0	0
male flowering	68	±3	51	±2	1363	960	1062	773
female flowering	73	±4	56	±3	1460	1072	1150	860
harvest	140	±3	129	±2	2951	2735	2277	2086
	**2023 (n = 250)**							
emergence	6	±1	6	±1	90	90	0	0
male flowering	73	±4	55	±2	1268	878	1042	727
female flowering	77	±5	62	±3	1358	1033	1113	845
harvest	153	±2	140	±2	3047	2772	2428	2208

**Table 7:** Greenhouse fertilization program.

In 1000 L Water	YaraTera Calcinit (15.5% N + 26.3% CaO)	YaraTera Kristalon Yellow (NPK13-40-13 + B, Cu, Fe, Mn, Mo and Zn)	YaraTera Kristalon White LB (NPK15-5-30 + Mg, S, B, Mo, Cu, Fe, Mn and Zn)	YaraTera Kristalon Brown (NPK3-11-38 + Mg, S, B, Cu, Fe, Mn, Mo and Zn)
1st–2nd week	240 g	240 g	120 g	-
3rd week	320 g	320 g	160 g	-
from flowering induction	90 g	180 g	90 g	540 g
7 days prior harvest	water deprivation			

## Data Availability

The original contributions presented in this study are included in the article. Further inquiries can be directed to the corresponding author.

## Supplementary Materials

The following supporting information can be downloaded at: https://www.mdpi.com/article/10.3390/plants14040594/s1, Table S1: correlation analyses of thousand kernel weight and seed number.

## Abbreviations

The following abbreviations are used in this manuscript:

VARI	Visible Atmospherically Resistant Index
UAV	Unmanned aerial vehicle
THC	Tetrahydrocannabinol
THCA	Tetrahydrocannabinolic acid
CBD	Cannabidiol
CBDA	Cannabidiolic acid
LED	Light-emitting diode
GDDs	Growing degree days
F1	First filial generation
EL×CH	Cross progeny of Eletta Campana female and Chamaeleon male
CH×CH	Cross progeny of Chamaeleon female and Chamaeleon male
BC1	First backcross progeny of the EL×CH female and Chamaeleon male
TLC	Thin layer chromatography
